# Comparison of the effects of remifentanil and dexmedetomidine on surgeon satisfaction with surgical field visualization and intraoperative bleeding during rhinoplasty

**DOI:** 10.1186/s12871-021-01546-9

**Published:** 2022-01-14

**Authors:** Reza Jouybar, Maryam Nemati, Naeimehossadat Asmarian

**Affiliations:** Anesthesiology and Critical Care Research Center, Shiraz University of Medical Sciences, Shiraz, 71937-11351 Iran

**Keywords:** Dexmedetomidine, Remifentanil, Rhinoplasty

## Abstract

**Objective:**

We aimed to compare the effect of dexmedetomidine with remifentanil on hemodynamic stability, surgical field quality, and surgeon satisfaction during rhinoplasty.

**Methods and materials:**

In this double-blind randomized controlled-trial, 60 participants scheduled for rhinoplasty at the Mother and Child Hospital, Shiraz, Iran, was randomely divided into the dexmedetomidine group (IV infusion of 1 μg/kg dexmedetomidine over 20 min before induction of anesthesia then 0.6 μg/kg/hr. dexmedetomidine from the time of induction until the end of the operation) or in the the remifentanil group (an infusion rate of 0.25 μg/kg/min from the time of anesthesia induction until the end of the operation). Bleeding volume, surgeon satisfaction, postoperative pain (visual analog scale (VAS)), Level of sedation (Richmond Agitation Sedation Scale (RASS)), Patient satisfaction, Vital signs & recovery, and the Aldrete Score (used to discharge the patients from recovery) were measured for all participants.

**Results:**

The patients in the dexmedetomidine group had less bleeding (*p* = 0.047) and shorter time to return of respiration, extubation, and the postoperative recovery time (*p* < 0.001). The surgeon satisfaction was higher in the dexmedetomidine group (*p* < 0.001). Patient satisfaction was significantly different between the two groups (*p* < 0.001). VAS scores, intaking paracetamol, and RASS score were significantly lower in the remifentanil group (*p* < 0.001). SBP, DBP, MAP, and heart rate were lower in dexmedetomidine group.

**Conclusion:**

Dexmedetomidine was associated with relatively stable hemodynamics, leading to decreased intraoperative bleeding, recovery time, and greater surgeon satisfaction and the level of consciousness in the recovery ward. However, painlessness and patient satisfaction were greater with the use of remifentanil.

**Trial registration:**

IRCT20141009019470N112.

## Introduction

Rhinoplasty is still one of the most common cosmetic surgeries in the world. The surgical procedure itself and hemodynamic instability in the patient cause intraoperative bleeding, which affects the quality of the surgical field, the intra- and post-operative complications, and the surgical outcome. Various drugs such as high-concentration volatile anesthetics, magnesium sulfate, remifentanil, clonidine, calcium channel blockers, tranexamic acid, intravenous nitroglycerin, and sodium nitroprusside have been evaluated to control blood pressure and decrease blood loss during surgery, thereby improving the surgical field quality [1–5].

Remifentanil hydrochloride is a potent short-onset and short-acting opioid with organ-independent metabolism. Due to the synergistic effects of remifentanil with other anesthetics, it can be used intraoperatively to induce mild to moderate hypotension and controlled bradycardia. Therefore, it has been used in various operations like rhinoplasty to reduce bleeding and improve hemodynamic stability [6–8].

Dexmedetomidine hydrochloride is a specific alpha−2 adrenoreceptor agonist that has intrinsic analgesic and sedative properties coupled with anxiolytic and sympatholytic effects. It minimizes the hemodynamic and neuroendocrine responses to anesthesia and surgery by suppressing the sympathetic tone. This hemodynamic stability can improve the surgical outcome as well as both patient and surgeon satisfaction. Dexmedetomidine accompanied by other anesthetics causes a controlled reduction in blood pressure and heart rate and improves the quality of the surgical field [3, 9–11].

Many investigations have evaluated the effect of dexmedetomidine or remifentanil in rhinoplasty surgery, however there was not any study to compare the effect of dexmedetomidine with remifentanil on surgical field quality and surgeon satisfaction during rhinoplasty. Therefore, we conducted this study to compare the effect of dexmedetomidine with remifentanil on hemodynamic stability, surgical field quality, and surgeon satisfaction during rhinoplasty.

## Material & Methods

### Study design

This double-blind randomized controlled trial was registered in the Iranian Registry of Clinical Trials (IRCT 20141009019470 N112, 01-04 − 2021) and was approved by the Ethics Committee of Shiraz University of Medical Sciences, Shiraz, Iran. The study was conducted on all patients aged 18 to 45 years with American Society of Anesthesiologists (ASA) grades I or II who were scheduled for rhinoplasty at the Mother and Child Hospital (affiliated to Shiraz University of Medical Sciences) between April 2021 to June 2021. The patients were enrolled if they provided informed consent once the study protocol was thoroughly explained to them. The exclusion criteria included a history of hepatic impairment, renal impairment (creatinine ≥2 mg/dL), allergy and hypersensitivity to the drugs used in the study (dexmedetomidine, remifentanil, or propofol), substance abuse or benzodiazepine addiction, excessive use of analgesics/non-steroidal anti-inflammatory drugs, diabetes mellitus, coagulation and bleeding disorders, use of anticoagulants, cerebrovascular diseases or accidents, cardiovascular disease, heart conduction disorders, morbid obesity (body mass index (BMI) > 40), a positive history of motion sickness, women who had a history of nausea and vomiting before menstruation.

### Sample size and randomization

The sample size was calculated using GPower software according to a previous study based on the bleeding score and bleeding severity variables. We assumed an effect size of 0.80*,* a power of 80%, a significance level of 5%, and a dropout rate of 10% [12]. Overall, 30 patients were randomly assigned to each group using block randomization with a block size of 6 (the blocks were extracted from www.sealedenvelope.com). By an individual who was independent to study. The randomization sequence was in sealed envelope. Also, generating the random allocation sequence, measurements, assigning participants to interventions were done by individuals who were blinded to study.

### Induction of anesthesia

All patients after entering the operating room were monitored by ECG, pulse oximetry, and non-invasive blood pressure (NIBP) monitoring. After anesthesia induction, patients were intubated. Induction of anesthesia was performed with 0.05 mg/kg midazolam, 2 μg/kg fentanyl, 0.15 mg/kg morphine, 2 mg/kg propofol, and 0.15 mg/kg cisatracurium. Anesthesia was maintained via the intravenous infusion of propofol (150 μg/kg/min for the first twenty minutes, then 120 μg/kg/min for the second twenty minutes, then 100 μg/kg/min for the remaining time of the operation). During the operation, all patients received a mixture of 50% oxygen and 50% nitrous oxide. In the dexmedetomidine group (D), the patients received an IV infusion of 1 μg/kg dexmedetomidine over 20 min before induction of anesthesia then 0.6 μg/kg/hr. dexmedetomidine from the time of induction until the end of the operation. In the remifentanil group (R), the patients received remifentanil at an infusion rate of 0.25 μg/kg/min (infusion pump) from the time of anesthesia induction until the end of the operation.

The end of the operation was defined as when the surgeon and the assistant surgeon performed the dressing and announced the completion of surgery. At this time, all anesthetics were discontinued and the patient was ventilated with 100% oxygen. After the return of respiratory signs or any movements, the patients were given neostigmine (0.06 mg/kg IV) and atropine (0.03 mg/kg IV). Once the patient’s tidal volume reached two-thirds of the expected value and the patient would obey orders, he or she was extubated. The bed of each patient was situated in the head-up (10 degrees) position. All patients were operated on by the same surgeon using an identical surgical procedure.

### Blindness

For injection of dexmedetomidine ahead of anesthesia induction, identical syringes (in equal volumes) containing dexmedetomidine and normal saline were prepared by a physician not involved in the research based on a table provided by the statistician. The syringes were delivered to the treating physician, who injected them at the specified time without knowing the type of drug.

For continuous infusion, dexmedetomidine and remifentanil were prepared by the external physician in 50 ml syringes according to the table provided by the statistician. Again, the identical syringes were delivered to the treating physician, who was blinded to the nature of the drug and administered it at the scheduled time. All patients and physicians were unaware of the type of drug and study group.

### Study measures

1. *Bleeding volume*: The amount of intraoperative bleeding was measured according to the volume of blood suctioned as well as the volume of blood on the blood-stained gauzes.
2. *Surgeon satisfaction with surgical field quality*: This was evaluated according to the surgeon’s opinion using the Likert scale below:
  Score 1 (very poor): Uncontrollable bleeding.
  Score 2 (poor): Severe bleeding requiring repeated suctioning, with the quality of the field collapsing immediately after suctioning.
  Score 3 (satisfactory): Moderate bleeding requiring intermittent suctioning.
  Score 4 (good): Partial bleeding, sometimes requiring suctioning; the quality of the surgical field was good.
  Score 5 (excellent): No bleeding/bloodless field; the surgery field was excellent.
3. *Postoperative pain*: The severity of the patients’ pain in the recovery room was evaluated using a visual analog scale (VAS). On this scale, zero was indicative of painlessness and ten represented the maximum amount of pain. Postoperative pain was measured at full awakening and then after 15, 30, and 45 min. In the case of mild pain (VAS = 1–3), paracetamol was given to the patient, while in the case of greater pain (VAS > 3), meperidine was administered with an initial dose of 25 mg, which was increased to up to 100 mg over two hours if required with respiratory monitoring.
4. *Level of sedation*: The level of sedation was evaluated with the Richmond Agitation Sedation Scale (RASS). This scale ranges from a score of −5 (unarousable) to +4 (intense agitation) (Table 1).

**Table 1:** The Richmond Agitation–Sedation Scale (RASS)

Score	Term	Description
+4	Combative	Overtly combative or violent; immediate danger to staff
+3	Very agitated	Pulls on or removes tube(s) or catheter(s) or has aggressive behavior toward staff
+2	Agitated	Frequent non-purposeful movement or patient-ventilator dyssynchrony
+1	Restless	Anxious or apprehensive but movements not aggressive or vigorous
0	Alert and calm	Spontaneously pays attention to caregiver
-1	Drowsy	Not fully alert, but has sustained (more than 10 s) awakening, with eye contact, to voice
-2	Light sedation	Briefly (less than 10 s) awakens with eye contact to voice
−3	Moderate sedation	Any movement (but no eye contact) to voice
−4	Deep sedation	No response to voice, but any movement to physical stimulation
−5	Unarousable	No response to voice or physical stimulation
5. *Patient satisfaction:* At the time of discharge from recovery, patient satisfaction was measured on a five-point scale as detailed below:
  Score 1: Very poor.
  Score 2: Poor.
  Score 3: Satisfactory.
  Score 4: Good.
  Score 5: Very good.
6. *Vital signs & recovery:* The systolic blood pressure (SBP), diastolic blood pressure (DBP), mean arterial pressure (MAP), and heart rate were measured before anesthesia induction, after induction, before intubation, after intubation, and then every 15 min until 75 min after entering recovery. Throughout the operation, the target MAP was 60–70 mmHg. In the case of a higher MAP leading to excessive bleeding in the surgical field, a nitroglycerin infusion was started at a dose of 0.5 μg/kg/min. If the arterial blood pressure fell to less than 30% of the baseline MAP, first an intravenous fluid infusion was used to correct the blood pressure, with a bolus ephedrine dose of 5 mg being administered if the former was insufficient. If the heart rate dropped to less than 44 beats per minute, atropine 0.6 mg was given as a bolus. All prescribed drugs were recorded.
  The time interval from the time of discontinuation of anesthetics to the return of spontaneous respiration, the interval from the time of discontinuation of anesthetics to the patient’s extubation, the duration of surgery, and the duration of recovery were recorded in both groups. An Aldrete Score of 9–10 was used to discharge the patients from recovery. The Aldrete Score is a medical scoring system for the measurement of post-anesthesia recovery; it includes activity, respiration, consciousness, blood circulation, and oxygen saturation (Table 2).

**Table 2:**
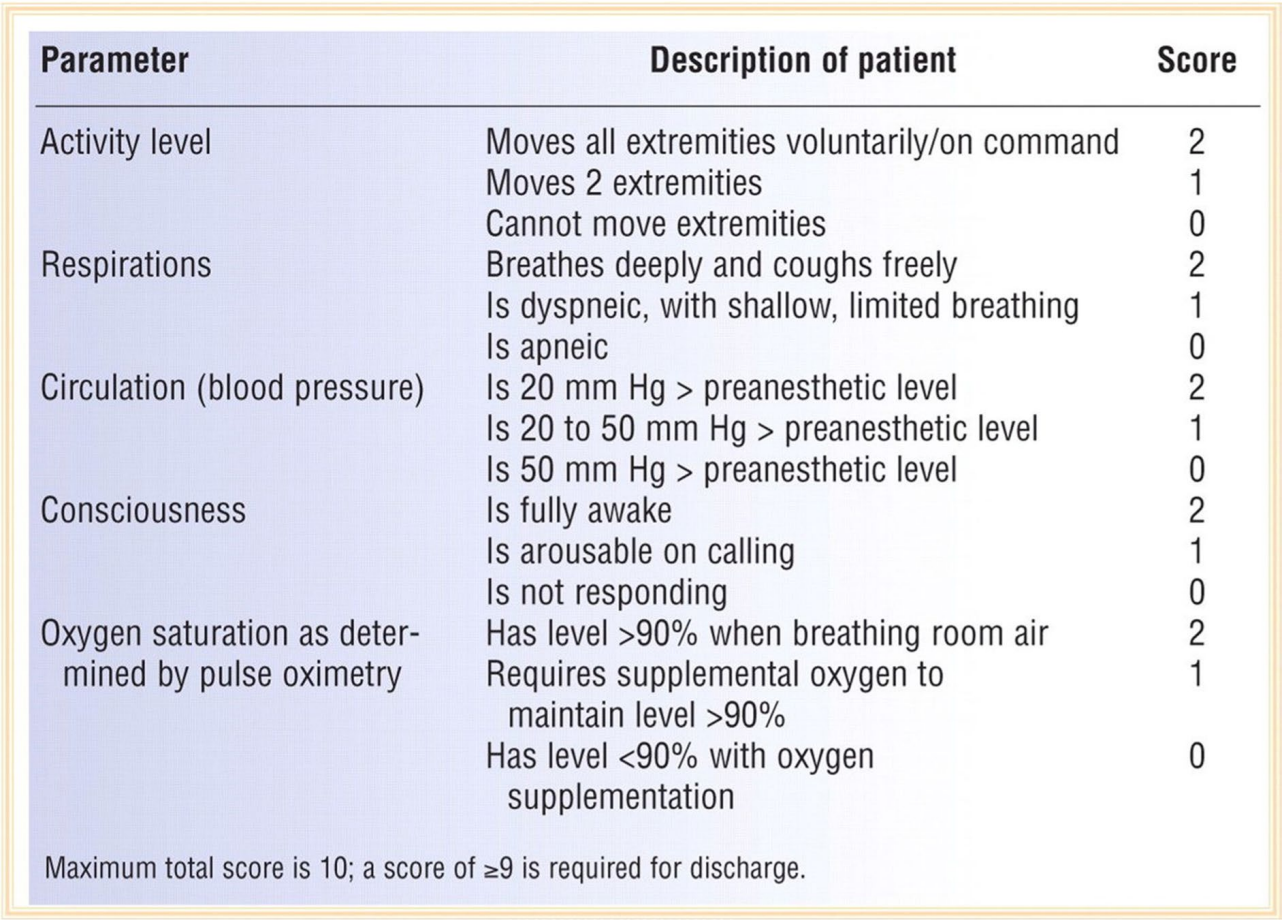
The Aldrete Score
  All mentioned data were recorded using a data collection form along with demographic data like age, gender, and weight.

### Data Analysis

In this study, continuous variables were reported as mean and standard deviation (SD). Categorical variables were reported as number and percentage. In order to compare the mean and median of quantitative variables between two groups, the independent sample t-test and Mann-Whitney test were used. Also, the chi-squared test and Fisher’s exact test were employed in the analysis of the categorical variables. As a significant interaction effect was noted in the repeated-measures analysis of variance (ANOVA) of the longitudinal dataset, univariate analysis was also done for data pertaining to each time point. Data were analyzed using SPSS 21 and *P*-values <0.05 were considered statistically significant.

## Results

**Fig. 1:**
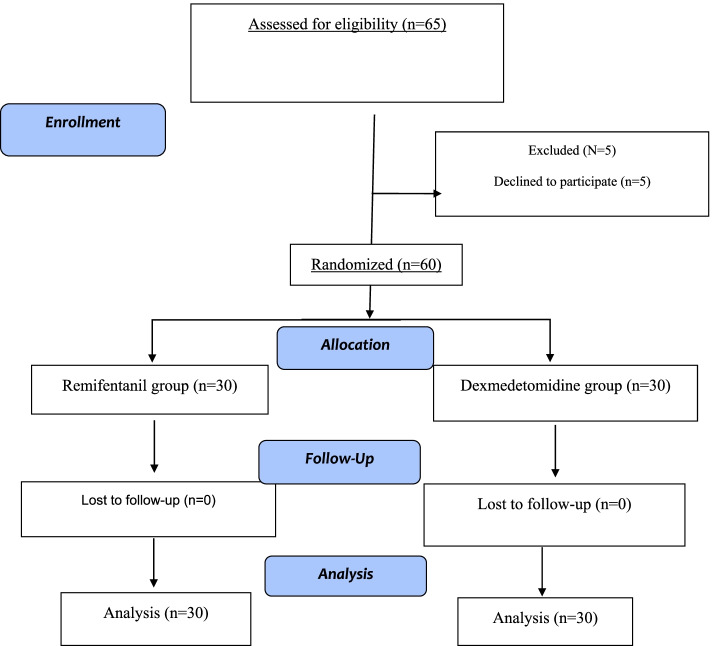
Patient enrollment and randomization flowchart

A total of 60 patients were considered in this study. The patients were randomly divided into the remifentanil and dexmedetomidine groups (Fig. 1).

The mean age of the patients was 30.19 ± 7.89 years (range: 17 to 46 years) and 52 of the patients were female. No significant differences were found between the groups in demographic and baseline data including age, sex, weight, and surgery time, indicating appropriate randomization (Table 3).

**Table 3:** Demographic and baseline data

Variable	Remifentanil (*n* = 30)	Dexmedetomidine B (*n* = 30)	*P*-value
Age, years	28.21 ± 8.01	32.10 ± 7.28	0.054
Sex, female	25 (83.3)	27 (90)	>0.999
Weight, kg	66.60 ± 5.65	70.00 ± 6.23	0.055
Surgery time, min	103 ± 2	96 ± 3	0.355

**Fig. 2:**
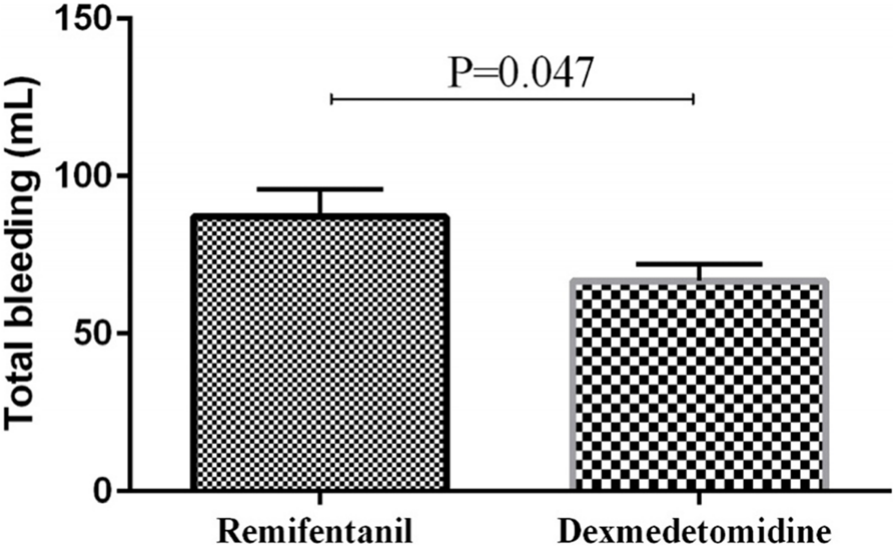
Comparing the amount of bleeding between the dexmedetomidine and remifentanil groups

The independent sample t-test showed that the patients in the dexmedetomidine group had less bleeding than those in the remifentanil group. The amount of bleeding in the dexmedetomidine group was 66.77 ± 5.35 ml compared to 92.75 ± 11.97 ml in the remifentanil group (*p* = 0.047) (Table 4, Fig. 2). The time until the return of spontaneous respiration, the time until extubation, and the postoperative recovery time were also lower in the dexmedetomidine group in comparison with the remifentanil group (*p* < 0.001) (Table 4).

**Table 4:** Comparison of studied variables between the dexmedetomidine and remifentanil groups

Variable		Remifentanil (***n*** = 30)	Dexmedetomidine (***n*** = 30)	***P***-value
Total bleeding, ml		92.75 ± 11.97	66.77 ± 5.35	0.047
Respiratory return time, min		15 (10–19)	5 (5–5)	<0.001
Extubation time, min		27 (20–30)	10 (10–15)	<0.001
Recovery time, min		35 (30–35)	22.5 (20–25)	<0.001
Surgeon satisfaction	Very poor	2 (6.9)	0 (0)	<0.001
	Poor	1 (3.4)	0 (0)	
	Fair	0 (0)	0 (0)	
	Good	15 (48.3)	1 (3.2)	
	Very good	12 (41.4)	29 (96.8)	
Patient satisfaction	Very poor	0 (0)	0 (0)	<0.001
	Poor	1 (3.4)	0 (0)	
	Satisfactory	4 (13.8)	6 (19.4)	
	Good	12 (37.9)	24 (80.6)	
	Very good	13 (44.8)	0 (0)	
RASS score		−1 (−1,-1)	1 (1–1)	<0.001
TNG, number (%)		4 (13.8)	0 (0)	<0.001
Paracetamol, number (%)		15 (50)	30 (100)	<0.001

*RASS* Richmond Agitation Sedation Scale, *TNG* Trinitroglycerin

Data pertaining to surgeon satisfaction during the operation is presented in Table 4. The satisfaction rate of the surgeon was higher in the dexmedetomidine group (*p* < 0.001). Surgeon satisfaction was ‘excellent’ in 96.8% of patients in the dexmedetomidine group and 41.4% of patients in the remifentanil group, and ‘good’ in 3.2% of patients in the dexmedetomidine group and 48.3% of patients in the remifentanil group. The surgeon did not express ‘very poor’, ‘poor’, or ‘fair’ satisfaction for any patient in the dexmedetomidine group, but had ‘very poor’ satisfaction in 6.9% of cases and ‘poor’ satisfaction in 3.4% of cases in the remifentanil group.

Patient satisfaction was significantly different between the two groups as well (*p* < 0.001). ‘Very poor’ satisfaction was not reported in either group. Notably, 82.7% of patients in the remifentanil group and 80.6% of patients in the dexmedetomidine group had good or very good satisfaction (Table 4).

A significant difference was observed between the groups regarding the RASS score (*p* < 0.001). The median RASS score in the remifentanil group was −1, compared to +1 in the dexmedetomidine group (Table 4).

Patients in the dexmedetomidine group did not receive TNG, but 13.8% of patients in the remifentanil group received TNG (*p* < 0.001) (Table 4).

In terms of pain, 50% of patients in the remifentanil group had no pain and therefore did not receive paracetamol, while all patients in the dexmedetomidine group received paracetamol (*p* < 0.001) (Table 4).

**Fig. 3:**
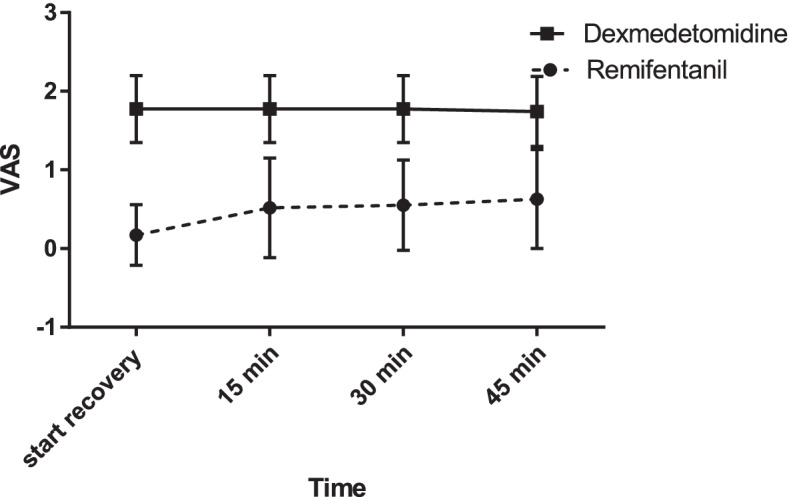
Comparing pain severity on the visual analog scale (VAS) between the dexmedetomidine and remifentanil groups

There was a time trend in pain in the remifentanil group. The VAS scores at 15, 30, and 45 min after entering the recovery room were significantly different (Fig. 3).

The Mann-Whitney test showed that VAS scores were significantly lower in the remifentanil group in comparison with the dexmedetomidine group at all time points (*p* < 0.001). The median VAS score in the remifentanil group was 0 in the recovery room at both 0 and 15 min, compared to 2 in the dexmedetomidine group (*p* < 0.001). At 30 and 45 min, the median VAS score was 1 in the remifentanil group and 2 in the dexmedetomidine group (Table 5).

**Table 5:** Comparing pain severity on the visual analog scale (VAS) between the dexmedetomidine and remifentanil groups

Variable	Remifentanil (***n*** = 30)	Dexmedetomidine (***n*** = 30)	***P***-value
VAS recovery start	0 (0–0)	2 (2–2)	<0.001
VAS 15 min	0 (0–1)	2 (2–2)	<0.001
VAS 30 min	1 (0–1)	2 (2–2)	<0.001
VAS 45 min	1 (0–1)	2 (1–2)	<0.001

The data were presents with median (IQR). *VAS* Visual analog scale

**Fig. 4:**
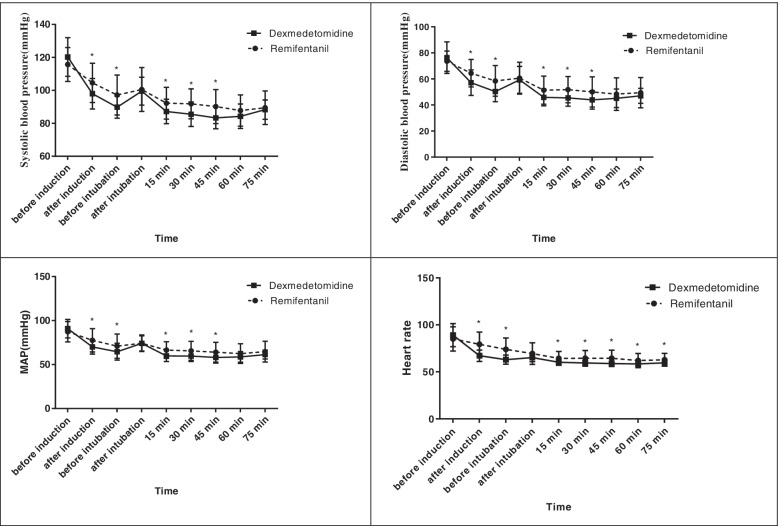
Comparing mean arterial pressure (MAP) and heart rate between the dexmedetomidine and remifentanil groups

The independent sample t-test showed that there was no difference among the two groups regarding SBP, DBP, and MAP before induction, after intubation, and at 60 and 70 min (*P* > 0.05). However, significant differences were observed after induction, just before intubation, and at 15, 30, and 45 min. The heart rate significantly differed at all time points except before induction and after intubation (Fig. 4).

## Discussion

In this study, the amount of bleeding in the dexmedetomidine group was less relative to the remifentanil group, leading to significantly higher surgeon satisfaction with the quality of the field of surgery and decreased frequency of suctioning. On the other hand, patient satisfaction was higher in the remifentanil group than in the dexmedetomidine group. However, the targeted hemodynamic goals were met to a greater in the dexmedetomidine group, which is why TNG was required in the remifentanil group but was not needed for any of the patients in the dexmedetomidine group. Patients in both groups had adequate analgesia, such that a pain score of above 3 on the VAS was never recorded and there was no need for narcotics (meperidine). Nonetheless, in the first minutes of entering the recovery ward, the degree of painlessness was less in the remifentanil group than in the dexmedetomidine group. According to the study protocol, paracetamol was injected for any patient who complained of mild pain (VAS less than 3). Therefore, due to the higher number of patients complaining of mild pain in the dexmedetomidine group, this group had greater painkiller use. Over time, the difference between the groups in this parameter decreased and the amount of painkiller consumption increased in the remifentanil group, though both painkiller use and pain score remained lower in the remifentanil group at all times. The RASS score was lower in the remifentanil group at the beginning of recovery, which probably explains the longer stay in recovery and longer time required to reach an Aldrete Score of 9–10 in patients in the dexmedetomidine group. On the other hand, the time until the return of spontaneous respiration and extubation was longer in the remifentanil group than in the dexmedetomidine group.

Bleeding is one of the most important and risky factors that affect the type and method of anesthesia chosen by surgeons and anesthesiologists. In the study of Bayram A et al., less bleeding and greater surgeon satisfaction were achieved using dexmedetomidine compared with magnesium sulfate among patients undergoing functional endoscopic sinus surgery. Furthermore, the amount of TNG use for controlling the blood pressure was also less in the dexmedetomidine group [13].

Qin Ye et al., in a study of patients undergoing laparoscopic cholecystectomy, found that using the right dose of dexmedetomidine gives rise to hemodynamic stability [14]. At high doses, dexmedetomidine inhibits the release of epinephrine and norepinephrine by stimulating presynaptic a2-adrenoreceptors and increased parasympathetic tone, thereby promoting hemodynamic stability [15, 16]. This mechanism probably explains the greater hemodynamic stability witnessed at all times in the dexmedetomidine group of our study relative to the remifentanil group. However, immediately following intubation, the efficacy of dexmedetomidine decreased leading to a peak in blood pressure and heart rate. This is probably due to the release of a large amount of epinephrine and norepinephrine following laryngoscopic stimulation and intubation, surpassing the neutralization capacity of dexmedetomidine at the dosage used. It should be noted that such a sudden rise in blood pressure and heart rate following intubation was also seen in the remifentanil group.

Hemodynamic stability is one of the most important factors that affect the level of bleeding and, consequently, the surgeon’s satisfaction with the operation. In the study of Somayaji A et al., which was performed on patients undergoing functional endoscopic sinus surgery, the researchers found that hemodynamic fluctuations were minimal, leading to increased surgeon satisfaction and better surgical outcomes [11]. Rokhtabnak F et al. demonstrated that during rhinoplasty, dexmedetomidine had a greater ability to stabilize hemodynamic parameters than magnesium sulfate, giving rise to greater surgeon satisfaction with the quality of the surgical field [17].

Pain and its complications can prolong the recovery time and maximize patient dissatisfaction. Therefore, various drugs are provided to reduce postoperative pain. The initial painlessness in the remifentanil group was probably due to the cumulative effects of the residual levels of narcotics and their metabolites (morphine and fentanyl), with the patients’ complaints of pain increasing gradually as the effect of the drugs disappeared and the RASS score increased [18]. On the other hand, the analgesic effects of dexmedetomidine are delivered through the hyperpolarization of interneurons along with a decrease in the release of the substance P and glutamate neurotransmitters, representing a weaker analgesic effect than that of remifentanil, which is in agreement with the findings of previous studies [15, 16].

Previous studies indicate that dexmedetomidine exerts its sedative effects by blocking presynaptic and post-synaptic a2-adrenergic receptors and that its mechanism of sedation, unlike drugs such as narcotics and benzodiazepines, is not through affecting the gamma-aminobutyric acid system. Patients are likely to be in an arousable and cooperative state when awakened from anesthesia, which averts delirium [15, 19, 20]. Considering that remifentanil suppresses respiration to a greater degree than dexmedetomidine, the time of the return of spontaneous respiration and also the time required for extubation was longer in the remifentanil group relative to the dexmedetomidine group [21–23]. In the study of Maud AS Weerink et al., it was found that the analgesic effect of dexmedetomidine was less than remifentanil up to the level of 2.4 ng/ml, and a targeted level of these drugs can be achieved using the mentioned systems [15].

In this study, there were many limitations, which could be eliminated through the use of larger sample sizes, Bispectral Index (BIS) monitoring, and a target-controlled infusion (TCI) system. This would improve the monitoring of the level of consciousness and sleep of the patients and would allow the measurement of drug use with greater accuracy.

## Conclusion

In this study, it was shown that dexmedetomidine was associated with relatively constant and stable hemodynamics, leading to decreased intraoperative bleeding and greater surgeon satisfaction relative to remifentanil. Although painlessness and patient satisfaction were greater with the use of remifentanil, the level of consciousness in the recovery ward was higher and the time until the return of spontaneous respiration, extubation, and discharge from recovery was shorter with the use of dexmedetomidine.

## Data Availability

All data will be available on request.

## References

[1] Gencay I, Muluk NB, Kilic R, Yazici I, Aydin G, Sencan Z (2020). Effects of Osteotomy on Hemodynamic Parameters and Depth of Anesthesia in Rhinoplasty Operations. J Craniofac Surg.

[2] Kosucu M, Tugcugil E, Arslan E, Omur S, Livaoglu MJ (2020). Effects of perioperative magnesium sulfate with controlled hypotension on intraoperative bleeding and postoperative ecchymosis and edema in open rhinoplasty. Am J Otolaryngol.

[3] Durmus M, But A, Dogan Z, Yucel A, Miman M, Ersoy MJ (2007). Effect of dexmedetomidine on bleeding during tympanoplasty or septorhinoplasty. Eur J Anaesthesiol.

[4] SJdA d V, do Nascimento-Júnior EM, de Aguiar Menezes MV, MLT M, de Souza Dantas R, PRSJ M-F (2018). Preoperative tranexamic acid for treatment of bleeding, edema, and ecchymosis in patients undergoing rhinoplasty: a systematic review and meta-analysis. JAMA Otolaryngol Head Neck Surg.

[5] Ghazipour A, Ahmadi K, Sarafraz M, Abshirini H, Akbari N (2013). Can clonidine as a pre-anaesthetic drug decrease bleeding during rhinoplasty surgery?. Indian J Otolaryngol Head Neck Surg.

[6] Servin FS, Billard V. Remifentanil and Other Opioids. In: Schüttler J., Schwilden H. (eds) Modern Anesthetics. Handbook of Experimental Pharmacology, vol 182. Berlin: Springer; 2008. 10.1007/978-3-540-74806-9_14. 10.1007/978-3-540-74806-9_14 18175097

[7] Mastronardi P, Cafiero T. Rational use of opioids. Minerva Anestesiologica. 2001;67(4):332–7. 11376535

[8] Kosucu M, Ömür S, Besir A, Uraloglu M, Topbas M, Livaoglu M (2014). Effects of perioperative remifentanil with controlled hypotension on intraoperative bleeding and postoperative edema and ecchymosis in open rhinoplasty. J Craniofac Surg.

[9] Parvizi A, Haddadi S, Habibi AF, Nemati S, Akhtar N, Ramezani H (2019). Dexmedetomidine efficacy in quality of surgical field during endoscopic sinus surgery. Iran J Otorhinolaryngol.

[10] Grape S, Kirkham K, Frauenknecht J, Albrecht EJ (2019). Intra-operative analgesia with remifentanil vs dexmedetomidine: a systematic review and meta-analysis with trial sequential analysis. Anaesthesia.

[11] Somayaji A, Raveendra US. Effect of dexmedetomidine on blood loss and quality of surgical field in functional endoscopic sinus surgery: a double blinded prospective controlled study. Karnataka Anaesthesia J. 2016;2(3):90–8.

[12] Modir H, Modir A, Rezaei O, Mohammadbeigi A (2018). Comparing remifentanil, magnesium sulfate, and dexmedetomidine for intraoperative hypotension and bleeding and postoperative recovery in endoscopic sinus surgery and tympanomastoidectomy. Med Gas Res.

[13] Bayram A, Ülgey A, Güneş I, Ketenci I, Çapar A, Esmaoğlu A (2015). Comparação entre dexmedetomidina e sulfato de magnésio em hipotensão controlada durante cirurgia funcional endoscópica dos seios paranasais.

[14] Ye Q, Wang F, Xu H, Wu L, Gao X (2021). Effects of dexmedetomidine on intraoperative hemodynamics, recovery profile and postoperative pain in patients undergoing laparoscopic cholecystectomy: a randomized controlled trial. BMC Anesthesiol.

[15] Weerink MA, Struys MM, Hannivoort LN, Barends CR, Absalom AR (2017). Colin PJCp. Clinical pharmacokinetics and pharmacodynamics of dexmedetomidine.

[16] Ebert TJ, Hall JE, Barney JA, Uhrich TD, Colinco MD (2000). The effects of increasing plasma concentrations of dexmedetomidine in humans. Anesthesiology.

[17] Rokhtabnak F, Motlagh SD, Ghodraty M, Pournajafian A, Delarestaghi MM, Banihashemi AT (2017). Controlled hypotension during rhinoplasty: A comparison of dexmedetomidine with magnesium sulfate. Anesth Pain Med.

[18] Romberg R, Olofsen E, Sarton E, den Hartigh J, Taschner PE, Dahan AJTJotASoA. (2004). Pharmacokinetic-pharmacodynamic modeling of morphine-6-glucuronide-induced analgesia in healthy volunteers: absence of sex differences. Anesthesiology.

[19] Segal IS, Vickery RG, Walton JK, Doze VA, Maze M. Dexmedetomidine diminishes halothane anesthetic requirements in rats through a postsynaptic alpha 2 adrenergic receptor. Anesthesiology. 1988;69(6):818–23. 10.1097/00000542-198812000-00004 2848424

[20] Maldonado JR, Wysong A, Van Der Starre PJ, Block T, Miller C, Reitz BAJP (2009). Dexmedetomidine and the reduction of postoperative delirium after cardiac surgery. Psychosomatics.

[21] Kaur M, Singh PJA (2011). Current role of dexmedetomidine in clinical anesthesia and intensive care. Anesth Essays Res.

[22] Venn RM, Hell J, Grounds RMJCC (2000). Respiratory effects of dexmedetomidine in the surgical patient requiring intensive care. Crit Care.

[23] Koo BN, Choi SH, Chun DH, Kil HK, Kim KJ, Min KT (2006). Respiratory depression caused by remifentanil infusion for postoperative pain control. Anesth Analg.

